# Biodegradable and Reusable Cellulose-Based Nanofiber Membrane Preparation for Mask Filter by Electrospinning

**DOI:** 10.3390/membranes12010023

**Published:** 2021-12-24

**Authors:** Jizhen Wang, Shaoyang Liu, Xu Yan, Zhan Jiang, Zijing Zhou, Jing Liu, Guangting Han, Haoxi Ben, Wei Jiang

**Affiliations:** 1 College of Textile and Clothing, Qingdao University, #308 Ningxia Road, Qingdao 266071, China; 18867319579@163.com (J.W.); qdyanx@163.com (X.Y.); kevinjiang_1987@126.com (Z.J.); jingj_l@126.com (J.L.); 2 Key Laboratory of Bio-Fibers and Eco-Textiles, Qingdao University, #308 Ningxia Road, Qingdao 266071, China; 3 Department of Chemistry and Physics, Troy University, Troy, AL 36082, USA; lius@troy.edu; 4 Shandong Special Nonwovens Engineering Research Center, Qingdao University, #308 Ningxia Road, Qingdao 266071, China; 5 Qingdao Xuyu Technology Co., Ltd., Qingdao 266071, China; j18812703738@163.com

**Keywords:** electrospinning, Cellulose, degradable, TPU, LiCl, air filtration

## Abstract

Environmentally friendly face masks with high filtration efficiency are in urgent need to fight against the COVID-19 pandemic, as well as other airborne viruses, bacteria and particulate matters. In this study, coaxial electrospinning was employed to fabricate a lithium chloride enhanced cellulose acetate/thermoplastic polyurethanes (CA/TPU-LiCl) face mask nanofiber filtration membrane, which was biodegradable and reusable. The analysis results show that the CA/TPU-LiCl membrane had an excellent filtration performance: when the filtration efficiency reached 99.8%, the pressure drop was only 52 Pa. The membrane also had an outstanding reusability. The filtration performance maintained at 98.2% after 10 test cycles, and an alcohol immersion disinfection treatment showed no effect on its filtration performance. In summary, the CA/TPU-LiCl nanofiber membrane made in this work is a promising biodegradable and reusable filtration material with a wide range of potential applications, including high-performance face mask.

## 1. Introduction

In the past a few decades, air pollution, especially particulate matters, has significantly intensified with the acceleration of industrialization, which has seriously affected people’s life quality and health [1]. Recently, the worldwide pandemic of COVID-19 seriously threatens human lives. More and more engineering measures and medical technologies are devoted to the treatment of particulates and the harmful substances they carry. Engineering measures such as dust reduction, dust suppression, dust capture and dust exhaust are restricted by places and occupations; COVID-19 still lacks specific therapeutic drugs, and mass vaccination requires a long period with high cost. Face masks have become the most effective and simple way to protect people from being invaded by viruses and airborne particles [2].

Melt-blown polypropylene (PP) non-woven fabric is widely applied to face mask manufacturing. However, it is difficult to control the fiber diameter and shape with the traditional preparation process. As a result, the fiber diameter could vary from 0.5 to 10 µm. The pore size of the filter material made with these fibers is not small enough to filter fine particles, such as COVID-19 viruses or aerosols, due to the relatively large and widely varied diameters of the fibers [3]. Therefore, electret treatment is required to improve the filtration efficiency when these fibers are used to manufacture high-efficiency masks. However, static electricity obtained from the electret treatment gradually dissipate during use and cleaning, significantly affecting the stability of the filter performance and the reusability of the face mask [4]. Nanofiber membrane prepared with electrospinning technology can achieve high filtration performance through small fiber diameter and pore size [5]. A large number of studies have reported electrospun fiber membranes used in the core layer of face masks [6,7,8,9,10,11]. Zhang et al. [6] prepared an anti-deformation PEO@PAN/PSU composite membrane through the combination of electrospinning technology and physical bonding process. The membrane showed a high filtration efficiency of 99.992%, a low pressure drop of 95 Pa, and an ideal quality factor of 0.1 Pa^−1^. Liu et al. [7] reported a bio-based polyamide 56 nanofiber/mesh (PA-56 NFN) membrane with bimodal structure for air filtration. When the pressure drop was 111 Pa, the filtration efficiency reached 99.995%. Yang et al. [8] prepared a PSA/PAN-B composite nanofiber-based filters by electrospinning, which showed high filtration efficiency (up to 99.52 ± 0.32%), low pressure drop (45.16 ± 1.39 Pa), excellent flexibility, good mechanical properties, high thermal stability (up to about 300 °C), and excellent chemical resistance. Although Jonas Matulevicius et al. [11] studied the filtration performance of cellulose acetate, they did not further optimize its filtration performance, and the best quality factor was only about 0.04.

Due to the protection needs of the pandemic, the massive production, use and disposal of face masks have caused serious environmental concerns. For example, in Wuhan, China, which has a population of more than 11 million, 200 tons of clinical waste, including a large number of discarded masks, were produced in a single day on 24 February 2020 [12]. It will take 450 years for the discarded face masks to decompose, and even if they decompose, they will cause serious damage to the ecosystem [13]. Therefore, it is urgent to develop biodegradable and reusable materials with high filtration efficiency for face masks [4]. Although the filter materials prepared by the researchers through the electrospinning method have excellent performance in air filtration, the processing and production of polymers will cause shortage of energy resources. In addition, the non-biodegradability of these polymers will also lead to environmental pollution and negative effects on human and animal health. Cellulose acetate as a derivative material of cellulose, its degradability has been confirmed by many studies [14,15].

In this work, a cellulose acetate (CA) based filtration membrane, which was biodegradable and reusable, was prepared with electrospinning technology. Its performance was optimized by adding thermoplastic polyurethane (TPU) and Lithium chloride (LiCl). The effects of fiber diameter on membrane pore size and filtration performance were investigated, reusability of the membrane was studied.

## 2. Experimental

### 2.1. Materials

Cellulose acetate (CA, acetyl 39.8%, hydroxyl 3.5%) powder was purchased from Shanghai Aladdin Chemical Co., Ltd., China. Thermoplastic polyurethane (TPU, 58887 polyether type) was obtained from American Noveon Chemical Co., Ltd. Lithium chloride (LiCl) was supplied by Shanghai Chemical Reagent Co., Ltd., Shanghai, China. Acetone (99.5%) and *N*,*N*-dimethylacetamide (DMAc, 99%) were received from Sinopharm Chemical Reagent Co., Ltd., Shanghai, China. PLA non-woven fabric (70 g/m^2^) was obtained from Hi-Tech Changjiang PLA Co., Ltd., Changshu, China. Wood pulp non-woven fabric (50 g/m^2^) was obtained from Jinkailong Technology Co., Ltd., Shenzhen, China. All materials and chemicals were used without further treatment.

### 2.2. Fabrication of Electrospun Fibrous Membranes

Cellulose acetate powder was placed in an acetone/DMAc mixture (mass ratio: 2:1) and stirred under room temperature on a magnetic agitator for 4–6 h until the CA powder was completely dissolved. Four spinning solutions with CA mass fractions of 10, 12, 14 and 16 wt% were obtained. SEM images of the filtration membranes made by the four CA solutions are shown in Figure S1. TPU elastomer was put in an acetone/DMAc mixture (mass ratio: 1:3) and stirred for 12 h with a magnetic stirrer to obtain four solutions with TPU mass fractions of 14, 16, 18 and 20 wt%, respectively. SEM images of the filtration membranes made by the four TPU solutions are shown in Figure S2. The above SEM images suggest that the best CA and TPU mass fractions are 14 wt% and 18 wt%, respectively. Therefore, the two concentrations were used in the rest part of the work. 

LiCl with a mass ratio of 0.5, 1, 2 and 3 wt% was dissolved in 14 wt% cellulose acetate solution to obtain CA-xLiCl solution. The CA-xLiCl solutions were mixed with the 18 wt% TPU solution at the mass ratio of 1:1, respectively, for single spinning. The CA-xLiCl solution and 18 wt% TPU were put in separated syringes, respectively, for coaxial spinning.

**Figure 1:**
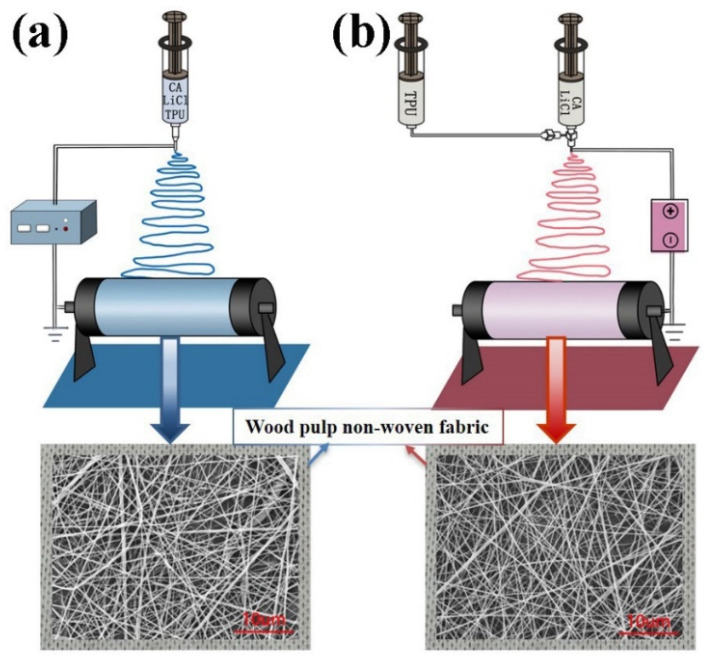
Schematic diagram of electrospinning techniques for fabricating nanofibers. (**a**) Single spinning, (**b**) Coaxial spinning.

For single spinning, an CA/TPU-xLiCl spinning solution was put into a 5 mL syringe. The advancing speed of the syringe of was set to 1 mL/h, the distance between the needle and the receiving drum was 18 cm, and the spinning voltage was 20 kV. The electrospun membrane was collected on the grounded roller at a rotating speed of 200 r/min with melt blown cloth (Figure 1a). The spinning was performed under a temperature of 27 ± 2 °C and a humidity of 40%–45%. The fabricated membranes were placed in an oven at 60 °C for 24 h to remove the residual solvent. The obtained CA/TPU-LiCl membrane fabricated with different concentrations of LiCl was denoted as CA/TPU-xLiCl. 

For coaxial spinning, one of the CA-xLiCl solutions and the 18 wt% TPU solution were put into two 5 mL syringes, respectively. The two syringes were placed on two injection pumps, respectively, and connected to a 22 G/17 G coaxial needle (Figure 1b). The forward speed of the two pumps was 0.5 mL/h. The other process parameters were the same to the single spinning.

### 2.3. Characterization

The morphology of the electrospun membrane was imaged by a scanning electron microscope (SEM, JSM-7008F, JEOL, Tokyo, Japan). The Nano Measurer software was used to measure the diameters of 50 randomly selected fibers to determine the average and distribution of the fiber diameter. The bubble point method was used to test the wet curve and dry curve of each membrane to obtain its pore size distribution with a pore size meter (TOPAS PSM165, Frankfurt, Germany). The electrical conductivity of spinning solution was measured by DDSJ-318 conductivity meter. Fourier transform infrared (FTIR) spectrum of each sample was obtained using a Nicolet iS10 (Thermo Fisher Scientific, Waltham, MA, USA). The spectra were acquired in the wavelength range of 250–4000 cm^−1^ with 32 scans and a resolution of 2 cm^−1^. Mechanical properties of membranes were measured by a strength tester (Instron 6025, Jinan Lianggong Testing Technology Co., Ltd., Jinan, China). The membranes were cut into a 50 × 20 mm rectangle for the mechanical tests. The initial clamping distance was 20 mm, and the stretching rate was 1 mm/min. The membrane thickness was measured using a thickness meter (YG141A, Wenzhou Jigao Inspection Instrument Co., Ltd., Wenzhou, China). Each sample was tested five times at random locations and the average value was taken, and input the measured thickness into the strength tester. Each membrane was cut into a circle with a diameter of 17 cm. The filtration efficiency and pressure drop of the membrane were tested by a filtration tester (TOPAS AFC-131, Frankfurt, Germany). The test particles were dioctyl sebacate (DEHS) aerosol particles generated by a polydisperse aerogel generator, and the concentration of the DEHS particles was 1.0 mg/m^3^. The air permeability of the membranes was tested by an air permeability tester (FX 3300, Text Test Instrument Company, Zurich, Switzerland) with a testing area of 200 cm^2^ and a pressure of 200 Pa.

### 2.4. Multiple Cycle Test and Disinfection Treatments

The durability of the filter membrane was characterized by 10 cycle filtration tests.

The fiber membrane and commercial mask were placed in a water bath and treated at 100 °C for 4 h. The fiber membrane and commercial mask were immersed in anhydrous ethanol for 4 h. The effects of high temperature and alcohol disinfection on the filtration performance of filter membrane and commercial mask were compared.

## 3. Results and Discussions

### 3.1. Morphology of Electrospun Fibers

**Figure 2:**
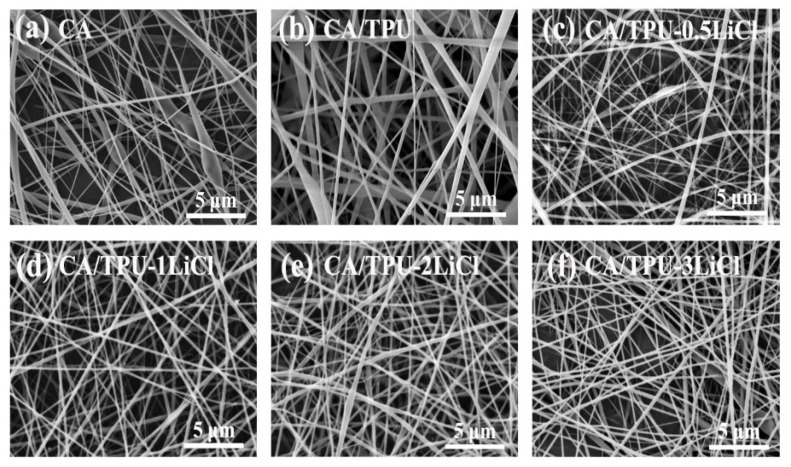
SEM images of (**a**) CA fibers, (**b**) CA/TPU fibers, and CA/TPU-xLiCl fibers prepared by the coaxial spinning with different LiCl concentrations: (**c**) 0.5 wt%, (**d**) 1 wt%, (**e**) 2 wt% and (**f**) 3 wt%.

The morphology and diameter distribution of the manufactured fibers are illustrated in Figure 2. Beads of different sizes were observed in the CA fibers (Figure 2a). This could be caused by the low viscosity of the 14 wt% CA solution, which leaded to insufficient entanglement between molecular chains and unstable jet during spinning. Meanwhile, due to the lack of elasticity of CA molecular chain, the external electric field could not fully stretch the molecular chains, which could also result in beads [16]. The introduction of TPU elastomer increased the viscosity of the mixed solution and the elasticity of molecular chains. Therefore, the degree of entanglement between the molecular chains increased, and the spinning solution could be stretched better by the electric field. As a result, beads largely disappeared in the prepared CA/TPU fibers [17]. When LiCl was added to the mixed solution, the electrical conductivity of the solution was improved, which improved the efficiency of the external electric field. So, the CA/TPU-xLiCl fibers prepared by the coaxial spinning were even more uniform than the CA/TPU fiber (Figure 2c–f).

### 3.2. Fiber Diameter and Pore Size of Membranes

**Figure 3:**
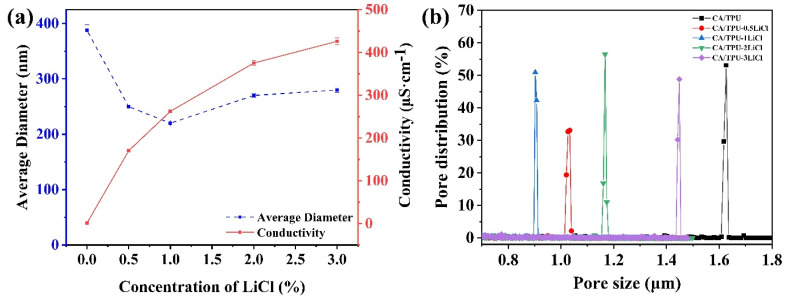
(**a**) Average diameter of the CA/TPU-xLiCl fibers and conductivity of the spinning solutions with different LiCl concentrations; (**b**) Pore size distribution of the CA/TPU-xLiCl membranes.

As shown in Figure 2 and Figure 3a, the diameter and uniformity of the fibers were notably affected by the concentration of LiCl. The average fiber diameter decreased from 390 nm to 220 nm as the concentration of LiCl increased from 0 to 1 wt% (Figure 3a). This was attributed to a larger Coulomb force caused by the increase of ion concentration. As a result, the spinning solution was stretching more under the external electric field, and finer fibers were formed [18]. When the concentration of LiCl further increased from 1 wt% to 3 wt%, thick knots appeared on the fibers, and the diameter increased from 220 nm to 280 nm (Figure 3a). This might be caused by the fluctuation of the spinning jet due to the excessive electrical conductivity (Figure 3a). The corresponding diameter distribution is shown in Figure S3. Comparing the uniformity and diameter of the CA/TPU-LiCl fibers, it could be concluded that the optimized LiCl concentration was 1 wt%.

Figure 3b shows the pore size distributions of filtration membranes formed by the CA/TPU-xLiCl fibers. Narrow distributions for all the membranes were observed. The average pore sizes of the membranes first decreased, but then increased with the increase of LiCl concentration from 0 to 3 wt%. The smallest average pore size of 0.90 μm was achieved when the LiCl concentration was 1 wt%, which confirms that the finest and most uniform fibers would form filtration members with the smallest pore size. Under this condition, more fibers can be piled up in the same space, the pore size of the fiber decreases with the increase of density, which is most suitable to intercept fine particles and achieve higher filtration efficiency [19].

### 3.3. Chemical Composition of the Membranes

**Figure 4:**
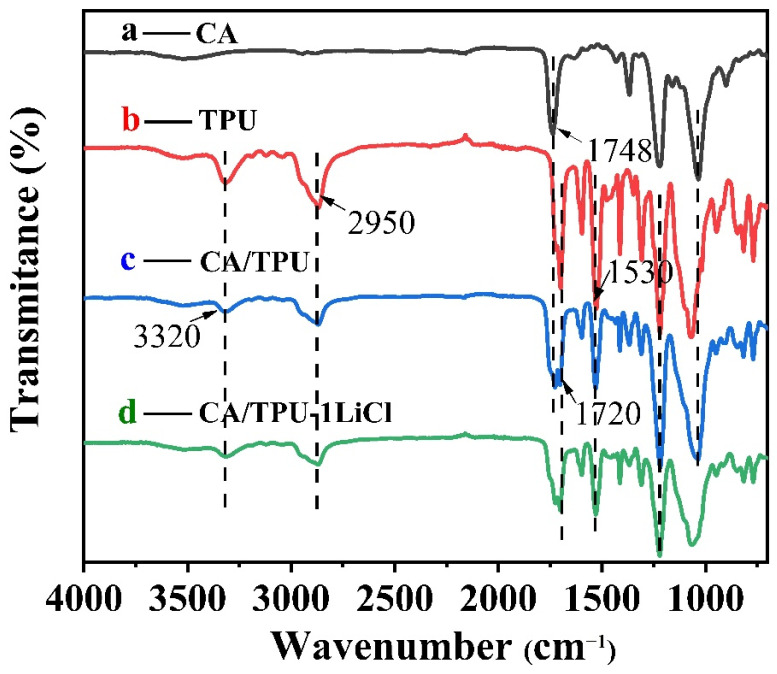
FTIR spectra of CA, TPU, CA/TPU and CA/TPU-1LiCl membranes.

The chemical composition of the membrane was monitored by infrared spectroscopy (Figure 4). In the infrared spectrum of the CA/TPU membrane, the absorption peak at 3320 cm^−1^ is attribute to the N-H or O-H stretching of TPU [20] and the peak at 2900–3000 cm^−1^ is assigned to the stretching of C-H in the methyl and methylene groups of TPU [21]. The absorption peaks at 1720 cm^−1^ and 1530 cm^−1^ correspond to the C = O stretching and N-H bending of TPU molecule, respectively [22]. The peak at 1748 cm^−1^ corresponds to the stretching of the ester carbonyl group in CA, which is characteristic absorption of the acetyl group [23]. The result confirms that the CA/TPU membrane contains both components. No new absorption band was observed in either the CA/TPU or CA/TPU-1LiCl spectra, indicating that the two polymers and LiCl were only physically mixed during the electrospinning process.

### 3.4. Mechanical Properties of the Membranes

**Figure 5:**
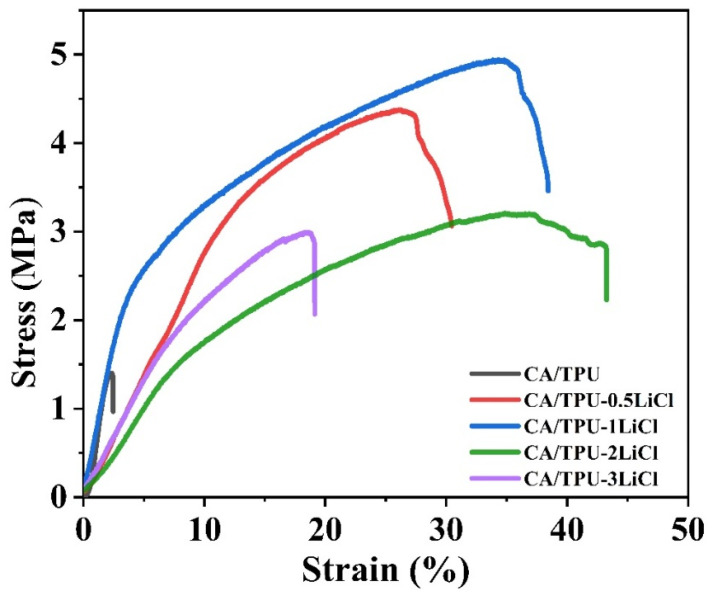
Stress-strain curves of each CA/TPU-xLiCl fiber membrane.

The stress–strain curves of the membranes are shown in Figure 5. When the LiCl concentrations were 0, 0.5 and 1 wt%, the tensile stress of the membranes was 1.4, 4.38 and 4.94 MPa, respectively. The increase of tensile stress was attribute to the more uniform fibers, which received evener force under the tensile test. When the LiCl concentration further increased to 2% and 3%, the tensile stresses dropped to 3.11 and 2.99 MPa, respectively. This was due to the unevenness of the fibers, which reduced the fiber packing density and increased the member thickness. In summary, the CA/TPU-1LiCl membrane showed the highest tensile stress, which was 3.5 times higher than that of the CA/TPU membrane. The improved mechanical property makes it more suitable to use as filtration material.

### 3.5. Evaluation of Filtration Performance of the Membranes

**Figure 6:**
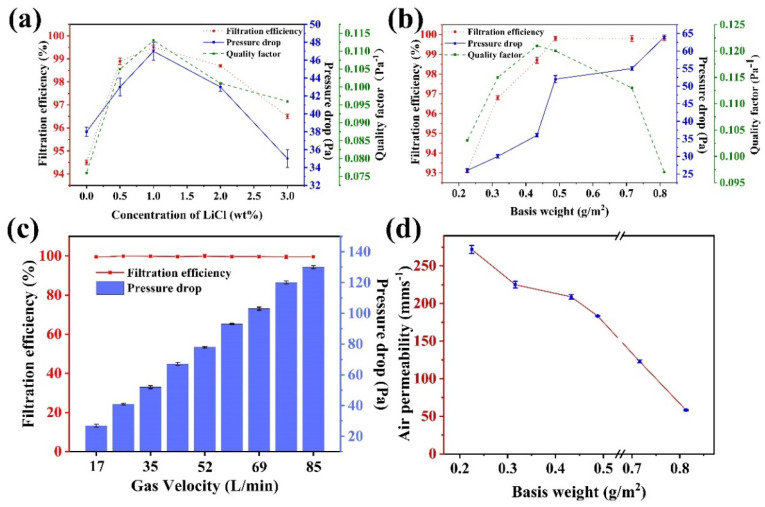
(**a**) Filtration performance of CA/TPU-xLiCl membrane under 35 L/min gas velocity; (**b**) Filtration performance of CA/TPU-1LiCl membrane with various basis weight under 35 L/min gas velocity; (**c**) Filtration performance of CA/TPU-1LiCl membrane under different gas velocity; (**d**) Air permeability of CA/TPU-1LiCl membranes with different gram weight.

The effects of LiCl concentration on filtration performance of the membranes are shown in Figure 6a. The filtration efficiencies of all membranes with LiCl were higher than that of the CA/TPU membrane. This could be attribute to the decreases of fiber diameter and membrane pore size with the addition of LiCl, which increased the probability of collision between the particles and the membranes. Since the CA/ TPU-1LiCl membrane had the most uniform fibers and the smallest pore size, it achieved the highest filtration efficiency of 99.5%. The filtration performance before and after the addition of LiCl was simulated in Figure S4.

Electrospun fibrous membranes are made of multiple layers of fibers. The filtration performance of the membrane is closely related to its basis weight. The higher the basis weight, the more fibers and, probably, the more layers of fibers present per unit area, and the better the filtration efficiency. Figure 6b shows the effect of basis weight on filtration performance of CA/TPU-1LiCl membrane. The filtration efficiency increased from 93.1% to 99.8% and the pressure drop increased from 26 Pa to 52 Pa when the basis weight of the membrane increased from 0.225 g/m^2^ to 0.475 g/m^2^. This is because the tighter and thicker packing of fibers provides better opportunities for fine particles to collide and adhere to the fibers, and, at the same time, increases the respiratory resistance [24]. When the basis weight of the membrane increased from 0.475 g/m^2^ to 0.83 g/m^2^, the pressure drop continued to rise to 109 Pa, but the filtration efficiency did not increase anymore. The effect of gram weight on the filtration performance of the fiber film was shown in Figure S5. As a face mask filtration material, in addition to filtration efficiency, pressure drop or airflow resistance is also an important parameter [25]. Its performance is evaluated with both filtration efficiency and pressure drop, namely, quality factor (QF, QF = −1n (1−η)/ΔP, where η and ΔP are the filtration efficiency and the pressure drop, respectively) [26]. As shown in Figure 6b, the maximum QF (0.12) under the gas velocity of 35 L/min was obtained when the CA/TPU-1LiCl membrane had a basis weight of 0.475 g/m^2^.

The filtration performance of the 0.475 g/m^2^ CA/TPU-1LiCl membrane at different gas velocities is presented in Figure 6c. The pressure drop increased gradually with the increase of gas velocity, but the filtration efficiency was maintained at 99.6%–99.8%. In addition, Figure S6 simulates the effect of changes in air velocity on the filtration efficiency and pressure drop of the fiber film. The results show that the prepared membrane can maintain high-efficiency filtration of micro-particles under different gas velocities.

Air permeability is an important parameter to evaluate the comfort performance of face masks. As illustrated in Figure 6d, there was a negative correlation between the basis weight of the membrane and the air permeability. This is because the thicker the fiber membrane is, the smaller the pores between the fibers are, and the less the air could pass. Testing under a certain pressure difference is not conducive to the rapid passage of airflow [27]. Therefore, the CA/TPU-1LiCl fiber membrane meets the requirements of commercial face mask standards for various purposes. This filtration material had both excellent filtration performance and relatively light weight.

Combined with Figure 6c and Table 1, it can be seen that the filtration performance of the materials prepared in this study can meet the requirements of relevant standards (GB 19083-2010 and T/CNTAC 55-2020). Compared with commercial masks, it has higher filtration efficiency with acceptable pressure drop value.

### 3.6. Reusability

The fiber membrane was subjected to a multiple cycle test, and alcohol and heating disinfection to test the reusability of the material and the effect of disinfection treatment on the filtration performance.

**Figure 7:**
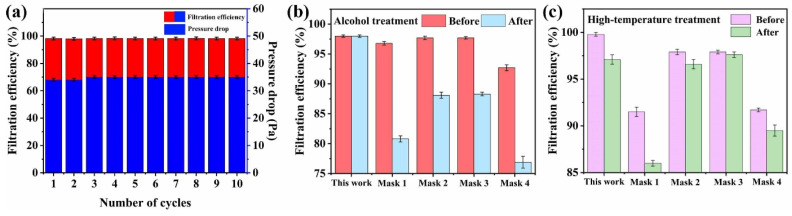
(**a**) Cycle test performance of the CA/TPU-1LiCl fiber membrane; (**b**) Effect of alcohol disinfection on filtration performance; (**c**) Effect of heating disinfection on filtration performance.

As shown in Figure 7, the filtration efficiency maintained at 98.2% after 10 cycles of tests, which only decreased 0.7%. The pressure drop was also stable at 34 Pa. The results indicate that the membrane was durable and could be reused at least 10 times.

The filter membrane and commercial masks were disinfected by alcohol soaking and heating to compare the effects of disinfection. The filtration efficiency of the CA/TPU-1LiCl membrane remained the same after the alcohol immersion treatment, while the filtration efficiency of commercial masks decreased significantly to various levels (Figure 7b). This might be because that the attenuation of the electret electric field of polypropylene melt blown nonwovens in the commercial masks due to the solvent immersion lowered the filtration efficiencies. After the heating treatment, the filtration efficiencies of the CA/TPU-1LiCl membrane and all commercial masks decreased notably (Figure 7c). The CA/TPU-1LiCl membrane could be affected by the thermal degradation of CA, while the commercial ones were affected by the reduced electret electric field. In summary, alcohol disinfection is more suitable for the CA/TPU-1LiCl membrane. Its stability was typically better than the commercial masks after disinfection treatments.

### 3.7. Selection of Inner and Outer Materials

The spunbond layer of the mask is usually made of polyester as a raw material. Considering the degradability of the mask, the design is optimized. Wood pulp and polylactic acid (PLA) are biodegradable materials, which are intended to be used instead of polyester nonwovens.

**Figure 8:**
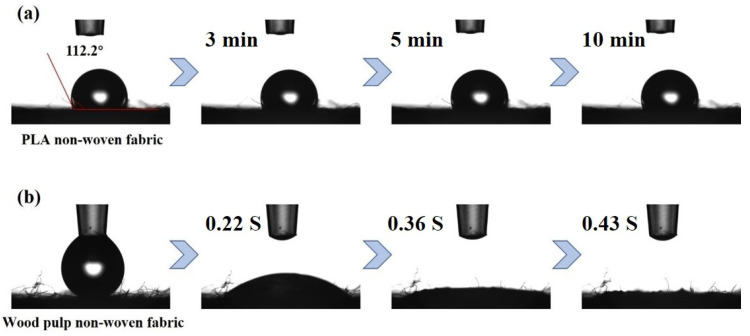
Contact angle of non-woven fabric (**a**) PLA, (**b**) Wood pulp.

As shown in Table 2, through the test of the filtration performance of the electrospinning membrane/non-woven composite membrane, it was concluded that the non-woven cloth had almost no contribution to the filtration performance of the composite membrane, and the filtration performance mainly depends on the electrospinning membrane. This was due to the great difference in diameter between non-woven and electrospinning membrane. As shown in Figure 8, PLA non-woven fabric was hydrophobic, and wood pulp non-woven fabric was hydrophilic. Considering the wettability of the material, PLA and wood pulp non-woven fabric were selected as the outer and inner materials of the mask, respectively.

## 4. Conclusions

A biodegradable and reusable cellulose-based face mask material was prepared in this work. Thin and even fibers were generated to form filtration membranes with small pore sizes, which enable the membranes to have excellent filtration performance. When the CA/TPU-1LiCl membrane reached the filtration efficiency of 99.8%, the pressure drop was only 52 Pa. In addition, cycle tests suggested that the membrane was stable and reusable. The alcohol disinfection treatment had no negative effect on the filtration performance of the material. This work not only developed a new promising face mask material, but also expanded the applications of cellulose acetate with electrospinning technology.

## Figures and Tables

**Table 1:** Comparison of filtration performance between CA/TPU-1LiCl fiber membrane and related mask standards and commercial masks.

Gas Velocity (L/min)	Filtration Efficiency (%)	Pressure Drop (Pa)	Quality Factor	Reference
35	99.8	52	0.12	This work
85	Level 1 ≥ 95 Level 2 ≥ 99 Level 3 ≥ 99.97	343.2		GB 19083-2010
30 ± 2	≥90	49		T/CNTAC 55-2020
35	91.5	10	0.247	Mask 1
35	97.7	22	0.17	Mask 2
35	97.7	32	0.118	Mask 3
35	91.70	22	0.113	Mask 4

**Table 2:** Effect of non-woven fabric and electrospinning membrane on filtration efficiency.

Materials	Filtration Efficiency (%)	Pressure Drop (Pa)	Quality Factor
PP non-woven fabric	29.3	1	0.35
Wood pulp non-woven fabric	33.8	4	0.1
PLA non-woven fabric	61.6	4	0.24
Electrospinning membrane/PP non-woven fabric	88.4	18	0.12
Electrospinning membrane/Wood pulp non-woven fabric	94.2	27	0.11
Electrospinning membrane/PLA non-woven fabric	92.1	23	0.11

## Data Availability

Data is contained within the article or Supplementary Materials.

## Supplementary Materials

The following supporting information can be downloaded at: https://www.mdpi.com/article/10.3390/membranes12010023/s1, Figure S1: SEM images of CA membranes with different mass fractions; Figure S2: SEM images of TPU membranes with different mass fractions; Figure S3: Diameter distribution of (a) CA fibers, (b) CA/TPU fibers, and CA/TPU-xLiCl fibers prepared by the coaxial spinning with different LiCl concentrations: (c) 0.5 wt%, (d) 1 wt%, (e) 2 wt% and (f) 3 wt%; Figure S4: Illustration showing the 3D model and dust particles filtration of CA/TPU and CA/ TPU-1LiCl films; Figure S5: 3D simulation of filtration efficiency and pressure drop of CA/ TPU-1LiCl fibrous films with different gram weight; Figure S6: 3D simulation of filtration efficiency and pressure drop of CA/ TPU-1LiCl fibrous film at different gas velocity.
