# Supplementary material for: Biodegradable and Reusable Cellulose-Based Nanofiber Membrane Preparation for Mask Filter by Electrospinning
Source: Membranes (Basel). 2021 Dec 24;12(1):23. doi: 10.3390/membranes12010023 (PMC8777847; doi:10.3390/membranes12010023)
Supplement: Supplementary file 1 [file membranes-12-00023-s001.zip › membranes-1499751-supplementary.pdf]

Supplementary information for

Biodegradable and reusable cellulose-based nanofiber membrane preparation for mask filter by electrospinning

Jizhen Wang [a,b], Shaoyang Liu [c], Xu Yan [a,d], Zhan Jiang [a,b], Zijing Zhou [e], Liu Jing [a] , Guangting Han [b,*], Haoxi Ben [b,*] and Wei Jiang [a,b,*]

a. College of Textile and Clothing, Qingdao University, #308, Ningxia Road, Qingdao, P.R. China.

b. Key Laboratory of Bio-Fibers and Eco-Textiles, Qingdao University, #308 Ningxia Road, Qingdao, P.R. China.

c. Department of Chemistry and Physics, Troy University, Troy, AL 36082, USA.

d. Shandong Special Nonwovens Engineering Research Center, Qingdao University, #308 Ningxia Road, Qingdao, P.R. China.

e. Qingdao Xuyu Technology Co., Ltd., Qingdao, P.R. China.

* Corresponding authors.

E-mail addresses: weijiangqd@qdu.edu.cn (Wei Jiang), kychgt@qdu.edu.cn (Guangting Han), benhaoxi@qdu.edu.cn (Haoxi Ben)

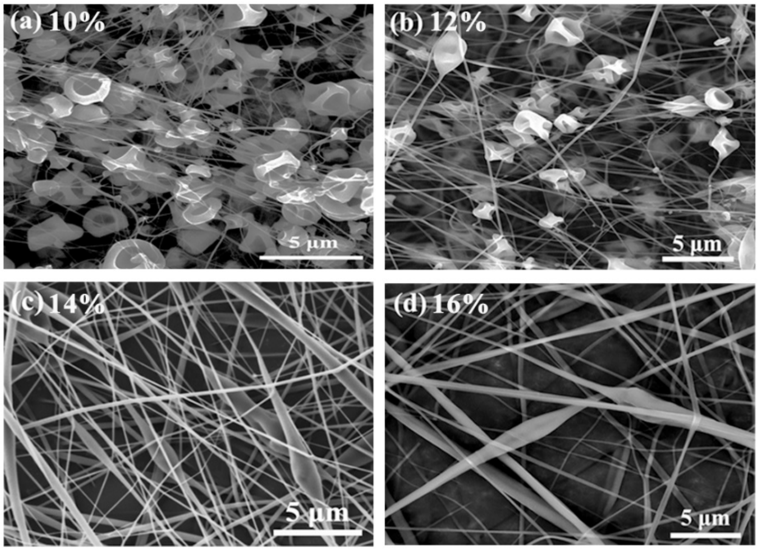

Figure S1. SEM images of CA membranes with different mass fractions.

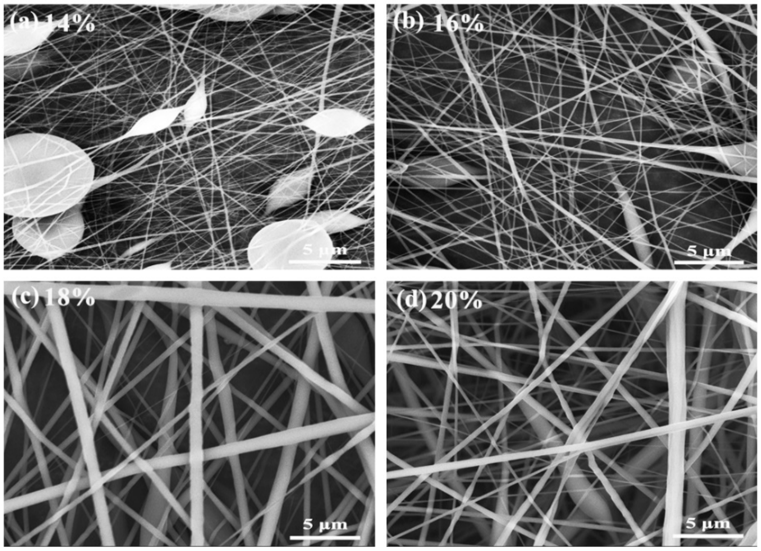

Figure S2. SEM images of TPU membranes with different mass fractions.

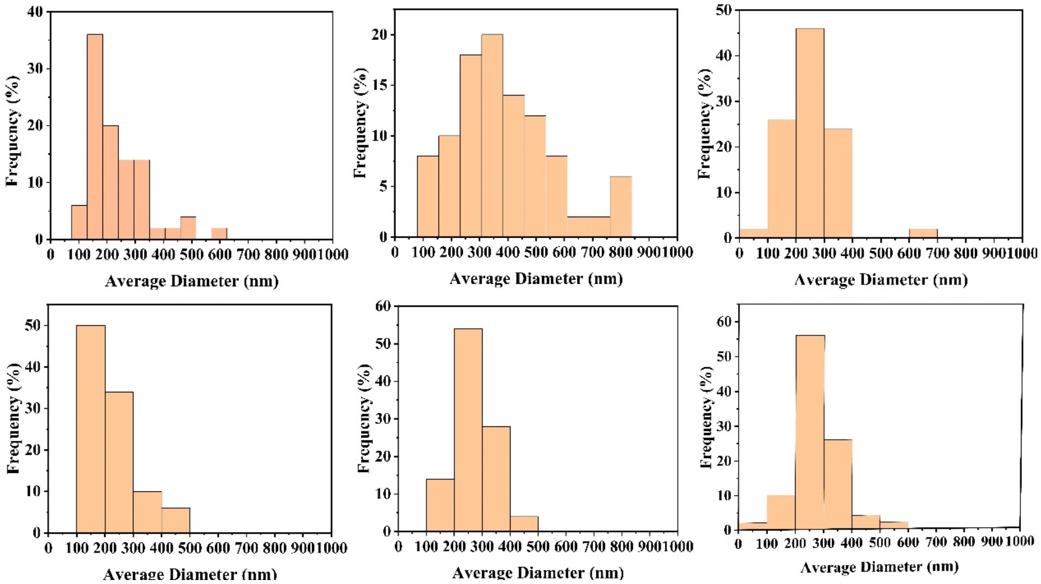

Figure S3. Diameter distribution of (a) CA fibers, (b) CA/TPU fibers, and CA/TPU-xLiCl fibers prepared by the coaxial spinning with different LiCl concentrations: (c) 0.5 wt%, (d) 1 wt%, (e) 2 wt% and (f) 3 wt%.

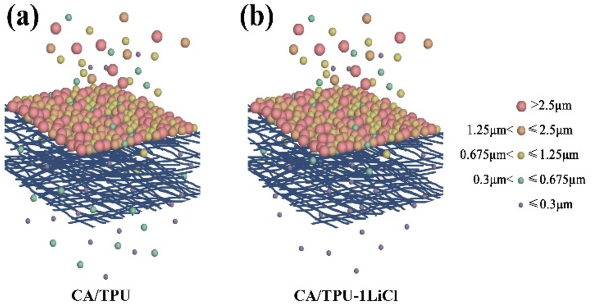

Figure S4. Illustration showing the 3D model and dust particles filtration of CA/TPU and CA/ TPU-1LiCl films

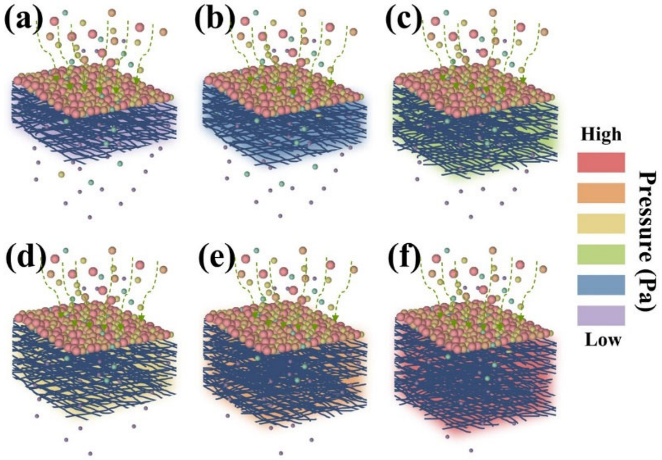

Figure S5. 3D simulation of filtration efficiency and pressure drop of CA/ TPU-1LiCl fibrous films with different gram weight.

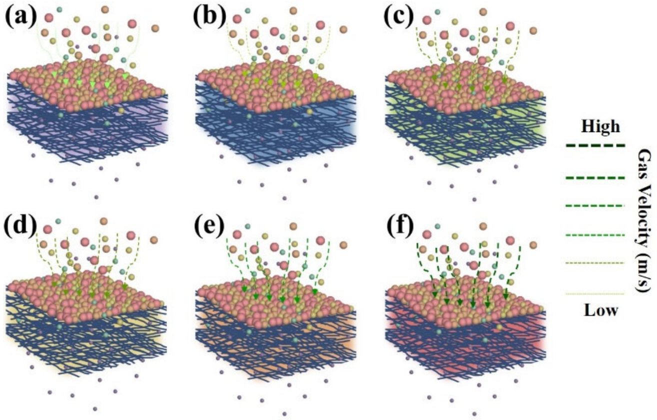

Figure S6. 3D simulation of filtration efficiency and pressure drop of CA/ TPU-1LiCl fibrous film at different gas velocity.
